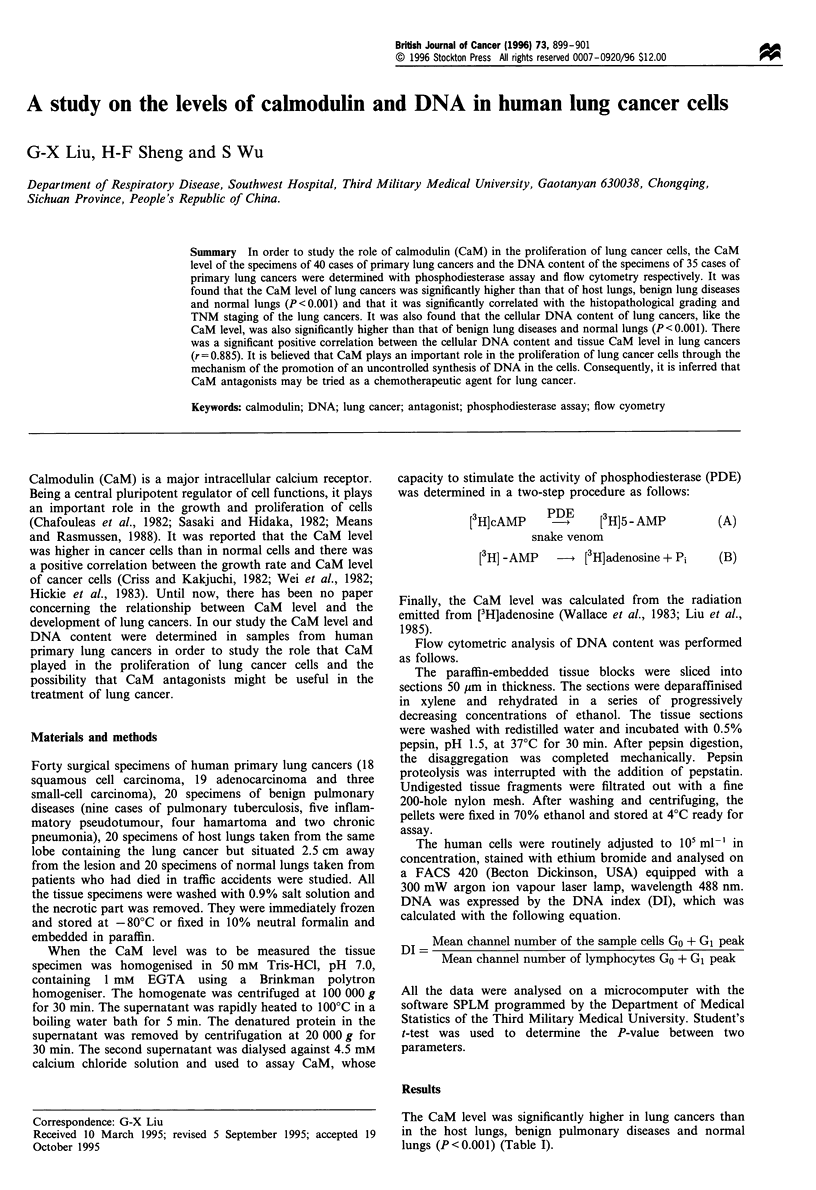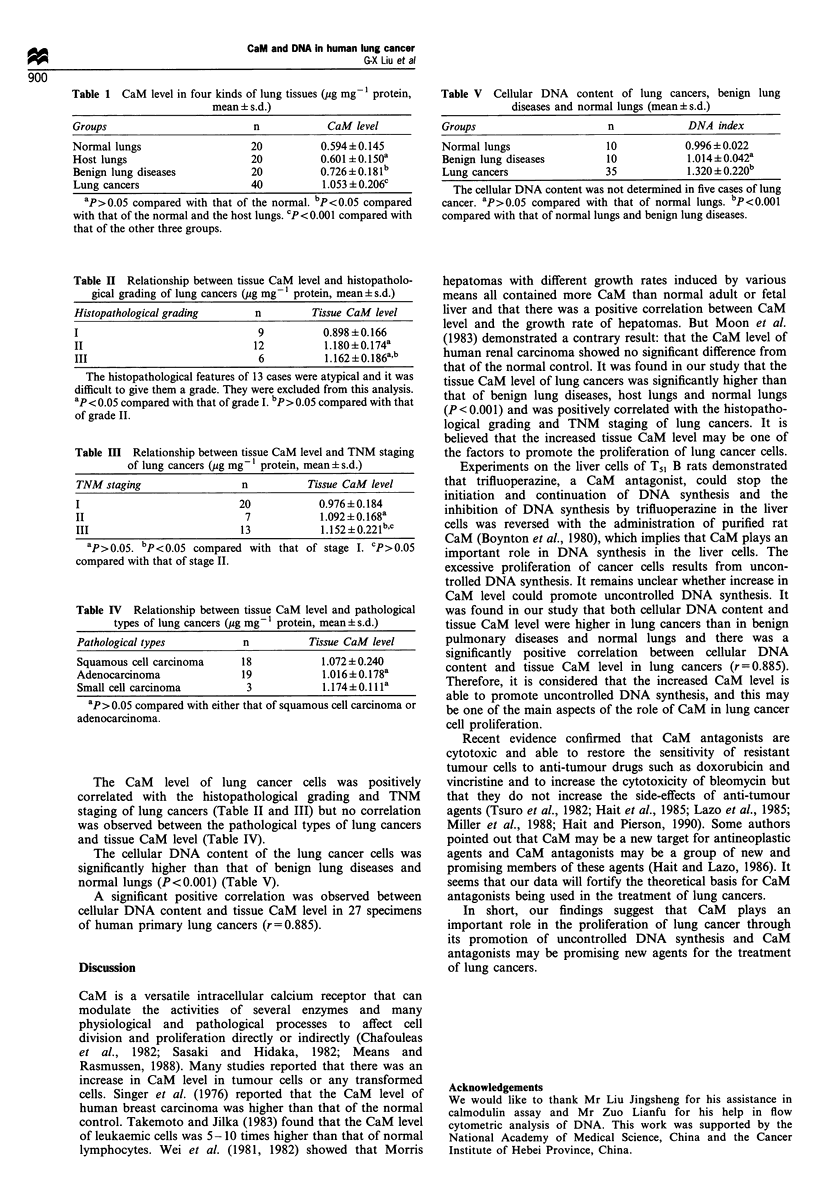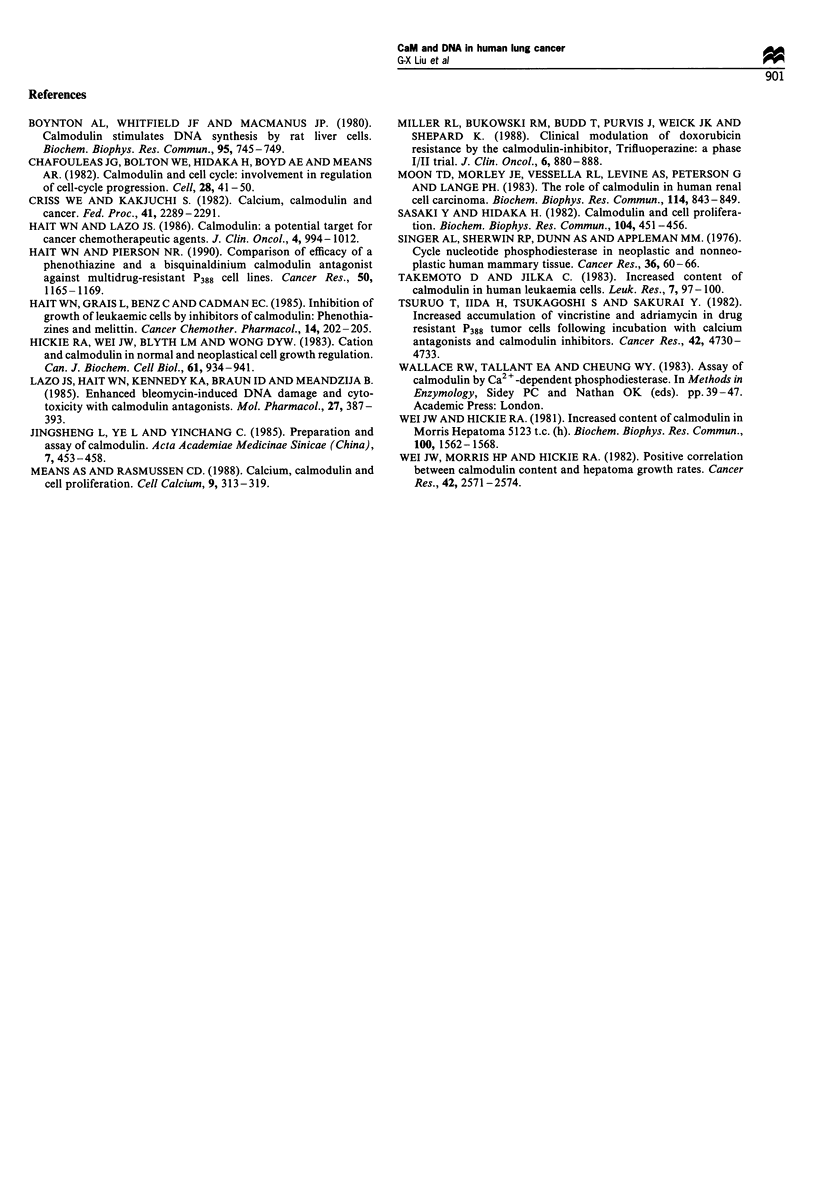# A study on the levels of calmodulin and DNA in human lung cancer cells.

**DOI:** 10.1038/bjc.1996.160

**Published:** 1996-04

**Authors:** G. X. Liu, H. F. Sheng, S. Wu

**Affiliations:** Department of Respiratory Disease, Southwest Hospital, Third Military Medical University, Gaotanyan, People's Republic of China.

## Abstract

In order to study the role of calmodulin (CaM) in the proliferation of lung cancer cells, the CaM level of the specimens of 40 cases of primary lung cancers and the DNA content of the specimens of 35 cases of primary lung cancers were determined with phosphodiesterase assay and flow cytometry respectively. It was found that the CaM level of lung cancers was significantly higher than that of host lungs, benign lung diseases and normal lungs (p<0.001) and that it was significantly correlated with the histopathological grading and TNM staging of the lung cancers. It was also found that the cellular DNA content of lung cancers, like the CaM level, was also significantly higher than that of benign lung diseases and normal lungs (p<0.001). There was a significant positive correlation between the cellular DNA content and tissue CaM level in lung cancers (r=0.885). It is believed that CaM plays an important role in the proliferation of lung cancer cells through the mechanism of the promotion of an uncontrolled synthesis of DNA in the cells. Consequently, it is inferred that CaM antagonists may be tried as a chemotherapeutic agent for lung cancer.


British Journal of Cancer (1996) 73, 899-901
© 1996 Stockton Press All rights reserved 0007-0920/96 $12.00

### A study on the levels of calmodulin and DNA in human lung cancer cells

G-X Liu, H-F Sheng and S Wu

*Department of Respiratory Disease, Southwest Hospital, Third Military Medical University, Gaotanyan 630038, Chongqing, Sichuan Province, People's Republic of China.*

**Summary** In order to study the role of calmodulin (CaM) in the proliferation of lung cancer cells, the CaM level of the specimens of 40 cases of primary lung cancers and the DNA content of the specimens of 35 cases of primary lung cancers were determined with phosphodiesterase assay and flow cytometry respectively. It was found that the CaM level of lung cancers was significantly higher than that of host lungs, benign lung diseases and normal lungs ($P<0.001$) and that it was significantly correlated with the histopathological grading and TNM staging of the lung cancers. It was also found that the cellular DNA content of lung cancers, like the CaM level, was also significantly higher than that of benign lung diseases and normal lungs ($P<0.001$). There was a significant positive correlation between the cellular DNA content and tissue CaM level in lung cancers ($r=0.885$). It is believed that CaM plays an important role in the proliferation of lung cancer cells through the mechanism of the promotion of an uncontrolled synthesis of DNA in the cells. Consequently, it is inferred that CaM antagonists may be tried as a chemotherapeutic agent for lung cancer.

**Keywords:** calmodulin; DNA; lung cancer; antagonist; phosphodiesterase assay; flow cyometry

Calmodulin (CaM) is a major intracellular calcium receptor. Being a central pluripotent regulator of cell functions, it plays an important role in the growth and proliferation of cells (Chafouleas *et al.*, 1982; Sasaki and Hidaka, 1982; Means and Rasmussen, 1988). It was reported that the CaM level was higher in cancer cells than in normal cells and there was a positive correlation between the growth rate and CaM level of cancer cells (Criss and Kakjuchi, 1982; Wei *et al.*, 1982; Hickie *et al.*, 1983). Until now, there has been no paper concerning the relationship between CaM level and the development of lung cancers. In our study the CaM level and DNA content were determined in samples from human primary lung cancers in order to study the role that CaM played in the proliferation of lung cancer cells and the possibility that CaM antagonists might be useful in the treatment of lung cancer.

### Materials and methods

Forty surgical specimens of human primary lung cancers (18 squamous cell carcinoma, 19 adenocarcinoma and three small-cell carcinoma), 20 specimens of benign pulmonary diseases (nine cases of pulmonary tuberculosis, five inflammatory pseudotumour, four hamartoma and two chronic pneumonia), 20 specimens of host lungs taken from the same lobe containing the lung cancer but situated 2.5 cm away from the lesion and 20 specimens of normal lungs taken from patients who had died in traffic accidents were studied. All the tissue specimens were washed with 0.9% salt solution and the necrotic part was removed. They were immediately frozen and stored at −80°C or fixed in 10% neutral formalin and embedded in paraffin.

When the CaM level was to be measured the tissue specimen was homogenised in 50 mM Tris-HCl, pH 7.0, containing 1 mM EGTA using a Brinkman polytron homogeniser. The homogenate was centrifuged at 100 000 *g* for 30 min. The supernatant was rapidly heated to 100°C in a boiling water bath for 5 min. The denatured protein in the supernatant was removed by centrifugation at 20 000 *g* for 30 min. The second supernatant was dialysed against 4.5 mM calcium chloride solution and used to assay CaM, whose capacity to stimulate the activity of phosphodiesterase (PDE) was determined in a two-step procedure as follows:

$$[^3H]cAMP \xrightarrow{PDE} [^3H]5\text{-}AMP \qquad (A)$$

$$[^3H]\text{-}AMP \xrightarrow{\text{snake venom}} [^3H]adenosine + P_i \qquad (B)$$

Finally, the CaM level was calculated from the radiation emitted from [$^3$H]adenosine (Wallace *et al.*, 1983; Liu *et al.*, 1985).

Flow cytometric analysis of DNA content was performed as follows.

The paraffin-embedded tissue blocks were sliced into sections 50 μm in thickness. The sections were deparaffinised in xylene and rehydrated in a series of progressively decreasing concentrations of ethanol. The tissue sections were washed with redistilled water and incubated with 0.5% pepsin, pH 1.5, at 37°C for 30 min. After pepsin digestion, the disaggregation was completed mechanically. Pepsin proteolysis was interrupted with the addition of pepstatin. Undigested tissue fragments were filtrated out with a fine 200-hole nylon mesh. After washing and centrifuging, the pellets were fixed in 70% ethanol and stored at 4°C ready for assay.

The human cells were routinely adjusted to $10^5$ ml$^{-1}$ in concentration, stained with ethium bromide and analysed on a FACS 420 (Becton Dickinson, USA) equipped with a 300 mW argon ion vapour laser lamp, wavelength 488 nm. DNA was expressed by the DNA index (DI), which was calculated with the following equation.

$$DI = \frac{\text{Mean channel number of the sample cells } G_0 + G_1 \text{ peak}}{\text{Mean channel number of lymphocytes } G_0 + G_1 \text{ peak}}$$

All the data were analysed on a microcomputer with the software SPLM programmed by the Department of Medical Statistics of the Third Military Medical University. Student's *t*-test was used to determine the *P*-value between two parameters.

### Results

The CaM level was significantly higher in lung cancers than in the host lungs, benign pulmonary diseases and normal lungs ($P<0.001$) (Table I).

Correspondence: G-X Liu
Received 10 March 1995; revised 5 September 1995; accepted 19 October 1995

**Table I** CaM level in four kinds of lung tissues ($\mu g\ mg^{-1}$ protein, mean ± s.d.)

| *Groups* | n | *CaM level* |
|---|---|---|
| Normal lungs | 20 | 0.594 ± 0.145 |
| Host lungs | 20 | 0.601 ± 0.150[a] |
| Benign lung diseases | 20 | 0.726 ± 0.181[b] |
| Lung cancers | 40 | 1.053 ± 0.206[c] |

[a]$P > 0.05$ compared with that of the normal. [b]$P < 0.05$ compared with that of the normal and the host lungs. [c]$P < 0.001$ compared with that of the other three groups.

**Table V** Cellular DNA content of lung cancers, benign lung diseases and normal lungs (mean ± s.d.)

| *Groups* | n | *DNA index* |
|---|---|---|
| Normal lungs | 10 | 0.996 ± 0.022 |
| Benign lung diseases | 10 | 1.014 ± 0.042[a] |
| Lung cancers | 35 | 1.320 ± 0.220[b] |

The cellular DNA content was not determined in five cases of lung cancer. [a]$P > 0.05$ compared with that of normal lungs. [b]$P < 0.001$ compared with that of normal lungs and benign lung diseases.

**Table II** Relationship between tissue CaM level and histopathological grading of lung cancers ($\mu g\ mg^{-1}$ protein, mean ± s.d.)

| *Histopathological grading* | n | *Tissue CaM level* |
|---|---|---|
| I | 9 | 0.898 ± 0.166 |
| II | 12 | 1.180 ± 0.174[a] |
| III | 6 | 1.162 ± 0.186[a,b] |

The histopathological features of 13 cases were atypical and it was difficult to give them a grade. They were excluded from this analysis. [a]$P < 0.05$ compared with that of grade I. [b]$P > 0.05$ compared with that of grade II.

**Table III** Relationship between tissue CaM level and TNM staging of lung cancers ($\mu g\ mg^{-1}$ protein, mean ± s.d.)

| *TNM staging* | n | *Tissue CaM level* |
|---|---|---|
| I | 20 | 0.976 ± 0.184 |
| II | 7 | 1.092 ± 0.168[a] |
| III | 13 | 1.152 ± 0.221[b,c] |

[a]$P > 0.05$. [b]$P < 0.05$ compared with that of stage I. [c]$P > 0.05$ compared with that of stage II.

**Table IV** Relationship between tissue CaM level and pathological types of lung cancers ($\mu g\ mg^{-1}$ protein, mean ± s.d.)

| *Pathological types* | n | *Tissue CaM level* |
|---|---|---|
| Squamous cell carcinoma | 18 | 1.072 ± 0.240 |
| Adenocarcinoma | 19 | 1.016 ± 0.178[a] |
| Small cell carcinoma | 3 | 1.174 ± 0.111[a] |

[a]$P > 0.05$ compared with either that of squamous cell carcinoma or adenocarcinoma.

The CaM level of lung cancer cells was positively correlated with the histopathological grading and TNM staging of lung cancers (Table II and III) but no correlation was observed between the pathological types of lung cancers and tissue CaM level (Table IV).

The cellular DNA content of the lung cancer cells was significantly higher than that of benign lung diseases and normal lungs ($P < 0.001$) (Table V).

A significant positive correlation was observed between cellular DNA content and tissue CaM level in 27 specimens of human primary lung cancers ($r = 0.885$).

### Discussion

CaM is a versatile intracellular calcium receptor that can modulate the activities of several enzymes and many physiological and pathological processes to affect cell division and proliferation directly or indirectly (Chafouleas *et al.*, 1982; Sasaki and Hidaka, 1982; Means and Rasmussen, 1988). Many studies reported that there was an increase in CaM level in tumour cells or any transformed cells. Singer *et al.* (1976) reported that the CaM level of human breast carcinoma was higher than that of the normal control. Takemoto and Jilka (1983) found that the CaM level of leukaemic cells was 5–10 times higher than that of normal lymphocytes. Wei *et al.* (1981, 1982) showed that Morris hepatomas with different growth rates induced by various means all contained more CaM than normal adult or fetal liver and that there was a positive correlation between CaM level and the growth rate of hepatomas. But Moon *et al.* (1983) demonstrated a contrary result: that the CaM level of human renal carcinoma showed no significant difference from that of the normal control. It was found in our study that the tissue CaM level of lung cancers was significantly higher than that of benign lung diseases, host lungs and normal lungs ($P < 0.001$) and was positively correlated with the histopathological grading and TNM staging of lung cancers. It is believed that the increased tissue CaM level may be one of the factors to promote the proliferation of lung cancer cells.

Experiments on the liver cells of $T_{51}$ B rats demonstrated that trifluoperazine, a CaM antagonist, could stop the initiation and continuation of DNA synthesis and the inhibition of DNA synthesis by trifluoperazine in the liver cells was reversed with the administration of purified rat CaM (Boynton *et al.*, 1980), which implies that CaM plays an important role in DNA synthesis in the liver cells. The excessive proliferation of cancer cells results from uncontrolled DNA synthesis. It remains unclear whether increase in CaM level could promote uncontrolled DNA synthesis. It was found in our study that both cellular DNA content and tissue CaM level were higher in lung cancers than in benign pulmonary diseases and normal lungs and there was a significantly positive correlation between cellular DNA content and tissue CaM level in lung cancers ($r = 0.885$). Therefore, it is considered that the increased CaM level is able to promote uncontrolled DNA synthesis, and this may be one of the main aspects of the role of CaM in lung cancer cell proliferation.

Recent evidence confirmed that CaM antagonists are cytotoxic and able to restore the sensitivity of resistant tumour cells to anti-tumour drugs such as doxorubicin and vincristine and to increase the cytotoxicity of bleomycin but that they do not increase the side-effects of anti-tumour agents (Tsuro *et al.*, 1982; Hait *et al.*, 1985; Lazo *et al.*, 1985; Miller *et al.*, 1988; Hait and Pierson, 1990). Some authors pointed out that CaM may be a new target for antineoplastic agents and CaM antagonists may be a group of new and promising members of these agents (Hait and Lazo, 1986). It seems that our data will fortify the theoretical basis for CaM antagonists being used in the treatment of lung cancers.

In short, our findings suggest that CaM plays an important role in the proliferation of lung cancer through its promotion of uncontrolled DNA synthesis and CaM antagonists may be promising new agents for the treatment of lung cancers.

**Acknowledgements**
We would like to thank Mr Liu Jingsheng for his assistance in calmodulin assay and Mr Zuo Lianfu for his help in flow cytometric analysis of DNA. This work was supported by the National Academy of Medical Science, China and the Cancer Institute of Hebei Province, China.

### References

BOYNTON AL, WHITFIELD JF AND MACMANUS JP. (1980). Calmodulin stimulates DNA synthesis by rat liver cells. *Biochem. Biophys. Res. Commun.*, **95**, 745–749.

CHAFOULEAS JG, BOLTON WE, HIDAKA H, BOYD AE AND MEANS AR. (1982). Calmodulin and cell cycle: involvement in regulation of cell-cycle progression. *Cell*, **28**, 41–50.

CRISS WE AND KAKJUCHI S. (1982). Calcium, calmodulin and cancer. *Fed. Proc.*, **41**, 2289–2291.

HAIT WN AND LAZO JS. (1986). Calmodulin: a potential target for cancer chemotherapeutic agents. *J. Clin. Oncol.*, **4**, 994–1012.

HAIT WN AND PIERSON NR. (1990). Comparison of efficacy of a phenothiazine and a bisquinaldinium calmodulin antagonist against multidrug-resistant $P_{388}$ cell lines. *Cancer Res.*, **50**, 1165–1169.

HAIT WN, GRAIS L, BENZ C AND CADMAN EC. (1985). Inhibition of growth of leukaemic cells by inhibitors of calmodulin: Phenothiazines and melittin. *Cancer Chemother. Pharmacol.*, **14**, 202–205.

HICKIE RA, WEI JW, BLYTH LM AND WONG DYW. (1983). Cation and calmodulin in normal and neoplastical cell growth regulation. *Can. J. Biochem. Cell Biol.*, **61**, 934–941.

LAZO JS, HAIT WN, KENNEDY KA, BRAUN ID AND MEANDZIJA B. (1985). Enhanced bleomycin-induced DNA damage and cytotoxicity with calmodulin antagonists. *Mol. Pharmacol.*, **27**, 387–393.

JINGSHENG L, YE L AND YINCHANG C. (1985). Preparation and assay of calmodulin. *Acta Academiae Medicinae Sinicae (China)*, **7**, 453–458.

MEANS AS AND RASMUSSEN CD. (1988). Calcium, calmodulin and cell proliferation. *Cell Calcium*, **9**, 313–319.

MILLER RL, BUKOWSKI RM, BUDD T, PURVIS J, WEICK JK AND SHEPARD K. (1988). Clinical modulation of doxorubicin resistance by the calmodulin-inhibitor, Trifluoperazine: a phase I/II trial. *J. Clin. Oncol.*, **6**, 880–888.

MOON TD, MORLEY JE, VESSELLA RL, LEVINE AS, PETERSON G AND LANGE PH. (1983). The role of calmodulin in human renal cell carcinoma. *Biochem. Biophys. Res. Commun.*, **114**, 843–849.

SASAKI Y AND HIDAKA H. (1982). Calmodulin and cell proliferation. *Biochem. Biophys. Res. Commun.*, **104**, 451–456.

SINGER AL, SHERWIN RP, DUNN AS AND APPLEMAN MM. (1976). Cycle nucleotide phosphodiesterase in neoplastic and nonneoplastic human mammary tissue. *Cancer Res.*, **36**, 60–66.

TAKEMOTO D AND JILKA C. (1983). Increased content of calmodulin in human leukaemia cells. *Leuk. Res.*, **7**, 97–100.

TSURUO T, IIDA H, TSUKAGOSHI S AND SAKURAI Y. (1982). Increased accumulation of vincristine and adriamycin in drug resistant $P_{388}$ tumor cells following incubation with calcium antagonists and calmodulin inhibitors. *Cancer Res.*, **42**, 4730–4733.

WALLACE RW, TALLANT EA AND CHEUNG WY. (1983). Assay of calmodulin by $Ca^{2+}$-dependent phosphodiesterase. In *Methods in Enzymology*, Sidey PC and Nathan OK (eds). pp. 39–47. Academic Press: London.

WEI JW AND HICKIE RA. (1981). Increased content of calmodulin in Morris Hepatoma 5123 t.c. (h). *Biochem. Biophys. Res. Commun.*, **100**, 1562–1568.

WEI JW, MORRIS HP AND HICKIE RA. (1982). Positive correlation between calmodulin content and hepatoma growth rates. *Cancer Res.*, **42**, 2571–2574.